# Supplementary material for: Novel Driver Strength Index highlights important cancer genes in TCGA PanCanAtlas patients
Source: PeerJ. 2022 Aug 11;10:e13860. doi: 10.7717/peerj.13860 (PMC9375969; doi:10.7717/peerj.13860)
Supplement: Supplemental Information 2 [file peerj-10-13860-s002.zip › COHORTS/genes plots/detailed/2021_8_16_14_9_distribution_events_detailed_females_1.pdf]

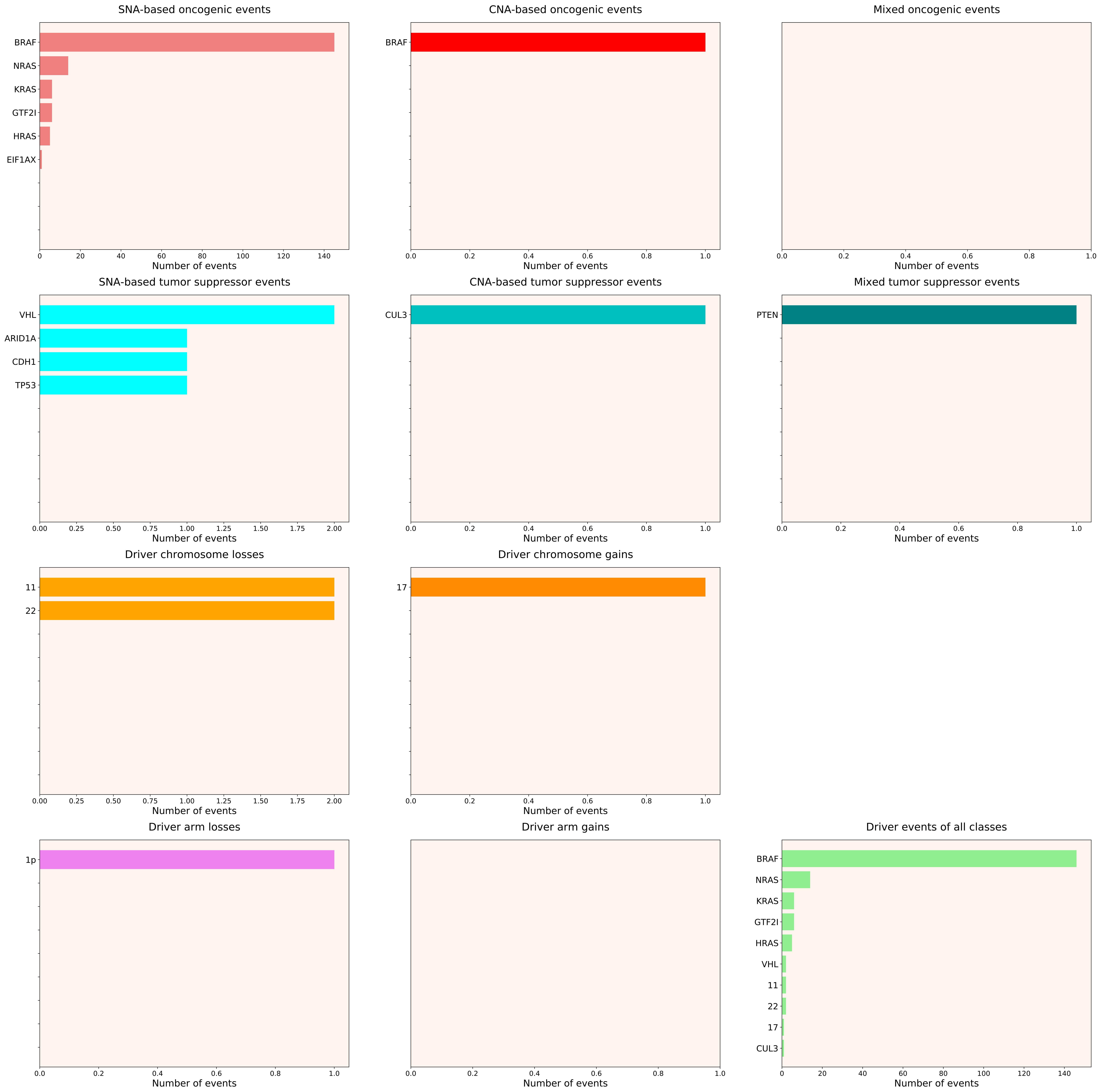
SNA-based oncogenic events
BRAF
NRAS
KRAS
GTF2I
HRAS
EIF1AX
0
20
40
60
80
100
120
140
Number of events
CNA-based oncogenic events
BRAF
0.0
0.2
0.4
0.6
0.8
1.0
Number of events
Mixed oncogenic events
0.0
0.2
0.4
0.6
0.8
1.0
Number of events
SNA-based tumor suppressor events
VHL
ARID1A
CDH1
TP53
0.00
0.25
0.50
0.75
1.00
1.25
1.50
1.75
2.00
Number of events
CNA-based tumor suppressor events
CUL3
0.0
0.2
0.4
0.6
0.8
1.0
Number of events
Mixed tumor suppressor events
PTEN
0.0
0.2
0.4
0.6
0.8
1.0
Number of events
Driver chromosome losses
11
22
0.00
0.25
0.50
0.75
1.00
1.25
1.50
1.75
2.00
Number of events
Driver chromosome gains
17
0.0
0.2
0.4
0.6
0.8
1.0
Number of events
Driver arm losses
1p
0.0
0.2
0.4
0.6
0.8
1.0
Number of events
Driver arm gains
0.0
0.2
0.4
0.6
0.8
1.0
Number of events
Driver events of all classes
BRAF
NRAS
KRAS
GTF2I
HRAS
VHL
11
22
17
CUL3
0
20
40
60
80
100
120
140
Number of events
